# The efficacy and safety of acupuncture for Parkinson’s disease insomnia: a systematic review and meta-analysis

**DOI:** 10.3389/fneur.2025.1697481

**Published:** 2025-11-03

**Authors:** Yujie Gu, Yue Liang, Hui Han, Huichao Yin, Zuncheng Zheng

**Affiliations:** ^1^Rehabilitation Centre, Taian Central Hospital, Taian, China; ^2^Rehabilitation Centre, Qingdao University Affiliated Taian Central Hospital, Taian, China; ^3^Rehabilitation Centre, Taishan Medical and Care Centre, Taian, China

**Keywords:** acupuncture, Parkinson’s disease, insomnia, systematic review, meta-analysis

## Abstract

**Background:**

Insomnia is a common comorbid symptom in Parkinson’s disease (PD) patients, significantly impairing their quality of life. Acupuncture is widely applied in treating PD insomnia, yet relevant evidence remains fragmented.

**Objective:**

To investigate the efficacy of acupuncture in improving PD insomnia through systematic review and meta-analysis, evaluating its clinical effectiveness and safety.

**Methods:**

Eight electronic databases were searched: PubMed, Cochrane Library, Embase, Web of Science, China National Knowledge Infrastructure (CNKI), VIP Data Platform, Wanfang Data Knowledge Service Platform, and China Biomedical Literature Service System. References from relevant literature and clinical trial registries were manually searched for randomized controlled trials (RCTs) on acupuncture for PD insomnia. Studies were screened against inclusion and exclusion criteria, relevant data extracted, and meta-analysis conducted using RevMan 5.4 software.

**Results:**

Eleven studies involving 800 patients were included. Meta-analysis revealed that acupuncture effectively improved PSQI (MD = −2.87, 95% CI: −4.28 to −1.46, *p* < 0.0001) and PDSS (MD = 7.96, 95% CI: 5.55–10.37, *p* < 0.00001), demonstrating superior efficacy compared to the control group (MD = 6.64, 95% CI: 3.47–12.69, *p* < 0.00001).

**Conclusion:**

Acupuncture effectively improves PSQI and PDSS scores in patients with PD insomnia and exhibits superior efficacy over the control group. However, due to limitations, further details could not be explored.

## 1 Introduction

Parkinson’s disease (PD) is a progressive, multisystem neurodegenerative disorder primarily affecting the elderly. Recent data indicate an increasing incidence of PD, making it the second most common neurodegenerative disease worldwide (1). Statistics reveal that PD currently affects over 6 million patients (2). With accelerating population aging, the incidence of PD is projected to rise further, doubling by 2050 (3, 4). In 1912, Frederick Lewy identified cytoplasmic inclusions (“Lewy bodies”) as pathological hallmarks of PD and discovered dopamine deficiency alongside its involvement in animal models of PD. Arvid Carlsson and Oleh Hornykiewicz subsequently established the link between dopamine deficiency and PD (5).

The motor symptoms of PD are typically characterized by bradykinesia, rigidity, and tremor resulting from degeneration of the dopaminergic system within the midbrain and basal ganglia (6). Non-motor symptoms encompass olfactory loss, constipation, and sleep disturbances (7). Among these, sleep disturbances constitute a primary non-motor symptom in PD patients, with insomnia being one of its principal manifestations (8, 9). Currently, diverse therapeutic approaches exist for managing insomnia in PD, Pharmacological interventions commonly employ benzodiazepines, non-benzodiazepine sedative-hypnotics, and melatonin receptor agonists. While these may provide short-term sleep improvement, long-term use carries risks of tolerance, dependence, and daytime somnolence. Furthermore, they may exacerbate PD motor symptoms or interact with dopaminergic medications (10–12). Non-pharmacological interventions such as cognitive behavioral therapy for insomnia (CBT-I) demonstrate efficacy, yet implementation among PD patients presents challenges. Some individuals struggle to complete full treatment courses due to motor impairment, cognitive decline, or emotional difficulties (13).

In recent years, acupuncture, as a traditional Chinese medical therapy, has demonstrated advantages in improving PD-related insomnia (14). Research indicates that acupuncture enhances sleep quality by modulating dopaminergic system function, inhibiting neuroinflammatory responses, and regulating the release of sleep-related neurotransmitters such as *γ*-aminobutyric acid (GABA) and serotonin within the brain (15–17). Several clinical trials have also reported that acupuncture significantly prolongs total sleep time and reduces nocturnal awakenings in PD patients, with fewer adverse reactions (18, 19). Compared to conventional pharmacological treatments, acupuncture offers holistic regulation, minimal side effects, and high patient acceptance, making it particularly suitable for elderly PD patients with multiple comorbidities and polypharmacy.

However, current evidence regarding acupuncture treatment for PD insomnia remains poorly synthesized, with a lack of high-quality evidence-based medical data to guide its clinical application. Therefore, this study conducted a systematic review and meta-analysis of randomized controlled trials investigating acupuncture for PD insomnia. It aimed to explore the efficacy and advantages of acupuncture in treating PD insomnia, providing reliable evidence for formulating clinical strategies and promoting the standardized application of acupuncture within the comprehensive management of PD insomnia.

## 2 Materials and methods

This study was analyzed in accordance with the PRISMA statement and was registered in PROSPERO prior to commencement (registration number: CRD420251112923).

### 2.1 Data sources and search strategy

This study searched eight electronic databases including PubMed, Cochrane Library, Embase, Web of Science, China Knowledge Infrastructure (CNKI), China Science Journal Database (VIP), Wanfang Database, and China Biomedical Literature Service System (CBM). In addition, we also manually searched the clinical registration platforms ClinicalTrials.gov and the Chinese ClinicalTrial Registry, as well as the reference lists of included studies and the references of relevant systematic reviews. Publications were restricted to English and Chinese language materials, with the search conducted up to 26 July 2025. A combination of MeSH terms and free-text keywords was employed, with primary search terms including “Parkinson Disease”, “Insomnia,” and “Acupuncture”. The specific search strategy is detailed in Table 1 (using PubMed as an example).

**Table 1:** Search strategy (PubMed as an example).

Rank	Search terms	Result
#1	Acupuncture[MeSH]	32,799
#2	Acupuncture Therapy[MeSH]	32,022
#3	Acupunctures, Ear[MeSH]	549
#4	Acupuncture Treatment OR Acupuncture Treatments OR Treatment, Acupuncture OR Therapy, Acupuncture OR Acupotomy OR Acupotomies OR Ear Acupunctures OR Auricular Acupuncture OR Ear Acupuncture OR Acupuncture, Auricular OR Acupunctures, Auricular OR Auricular Acupunctures OR Electroacupuncture OR Scalp Acupuncture OR Tongue Acupuncture	44,868
#5	#1 OR #2 OR #3 OR #4	45,095
#6	Parkinson Disease[MeSH]	91,055
#7	Parkinson Disease, Secondary[MeSH]	6,901
#8	Parkinsonian Disorders[MeSH]	107,664
#9	Idiopathic Parkinson’s Disease OR Lewy Body Parkinson’s Disease OR Parkinson’s Disease, Idiopathic OR Parkinson’s Disease, Lewy Body OR Parkinson Disease, Idiopathic OR Parkinson’s Disease OR Idiopathic Parkinson Disease OR Lewy Body Parkinson Disease OR Primary Parkinsonism OR Parkinsonism, Primary OR Paralysis Agitans	162,299
#10	#6 OR #7 OR #8 OR #9	168,568
#11	Sleep Initiation and Maintenance Disorders[MeSH]	20,346
#12	Dyssomnias[MeSH]	91,220
#13	Sleep Wake Disorders[MeSH]	120,474
#14	Sleep Disorders, Circadian Rhythm[MeSH]	2,968
#15	Sleeplessness OR Insomnia Disorder* OR Insomnia* OR Early Awakening OR Awakening, Early OR Nonorganic Insomnia OR Insomnia, Nonorganic OR Psychophysiological Insomnia OR Insomnia, Psychophysiological OR Secondary Insomnia OR Insomnia, Secondary OR Sleep Initiation Dysfunction OR Dysfunction?, Sleep Initiation OR Sleep Initiation Dysfunctions	47,500
#16	#11 OR #12 OR #13 OR #14 OR #15	142,033
#17	#5 AND #10 AND #16	19

### 2.2 Inclusion criteria and exclusion criteria

The inclusion criteria for this study were established according to the framework of PICOS (Population, Intervention, Comparison, Outcome, and Study Design). The specific inclusion criteria are as follows: (1) Subjects must be patients with a confirmed diagnosis of PD-related insomnia; (2) The intervention group received acupuncture ± conventional medication; (3) The control group was either treated with conventional drugs or a blank control; (4) The study design must be a randomized controlled trial.

Exclusion criteria: (1) Studies where subjects had unclear diagnoses or concomitant conditions affecting outcome assessment; (2) Studies involving concurrent interventions; (3) Studies with non-representative outcome measures; (4) Non-randomized controlled trials; (5) Studies where full texts were unavailable or data were incomplete.

### 2.3 Literature management and data extraction

Two researchers independently screened the literature and extracted data according to inclusion and exclusion criteria. Any discrepancies during this process were resolved through consultation with a third researcher. The specific screening procedure was as follows: (1) Duplicate records were excluded using the literature management software Endnote X9.3. (2) Titles and abstracts were reviewed to exclude review articles, theses, conference papers, scientific achievements, and other non-relevant literature. (3) Full-text reading to determine eligibility against inclusion criteria.

Following literature screening, the following information was extracted:

1. Study details: author information, publication year, etc.;
2. Subject characteristics: sample size, age, gender, diagnostic criteria, disease duration, etc.;
3. Intervention method, frequency, and duration;
4. Outcome measures.

### 2.4 Evaluation of literature quality

We employed the Cochrane Risk of Bias tool (ROB 2.0) to assess the quality of the included studies. This assessment tool comprises five domains: Bias in the randomization process, bias in deviation from the specified intervention, bias in missing outcome data, bias in outcome measurement, and bias in selective reporting of results. The bias in deviation from the specified intervention domain is further subdivided into two scenarios based on research objectives: one concerning the effect of intervention allocation, and the other concerning the effect of intervention adherence. Each domain contains multiple distinct signal questions. When assessing the risk of bias in RCTs, researchers must make judgments and objectively answer these questions. Signal questions typically offer five response options: Yes (Y), Probably Yes (PY), Probably No (PN), No (N), and No Information (NI).

### 2.5 Statistical analysis

In accordance with the PRISMA guidelines, statistical analyses were conducted using Review Manager 5.4.1, reporting pooled risk ratios (RR) and mean differences (MD) with 95% confidence intervals (CI). Statistical heterogeneity was quantified using the I^2^ statistic. Heterogeneity was defined as low, moderate, or high based on I^2^ values of 25, 50, and 75%, respectively. Publication bias for primary outcomes was visually assessed via funnel plots. Furthermore, owing to the limited number of studies per outcome (fewer than 10), we excluded the application of Egger’s regression test in the analysis of publication bias.

## 3 Results

### 3.1 Search results

**Figure 1:**
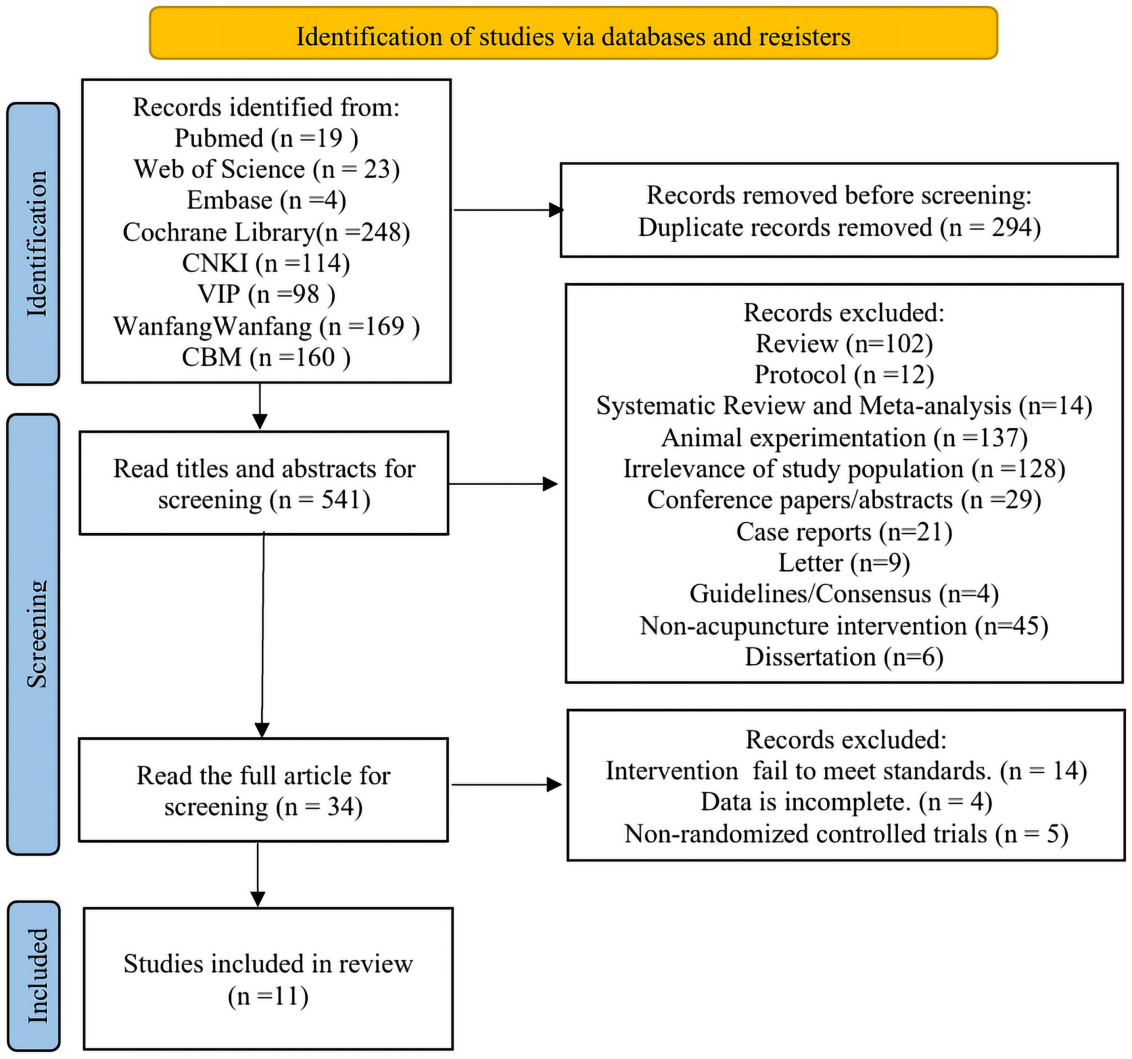
Literature screening process.

A total of 835 articles were retrieved, with 541 remaining after excluding duplicates. Following further screening based on titles and abstracts, 34 articles remained. After reviewing the full texts of these 34 articles, 11 ultimately met the inclusion criteria. The specific literature screening process is illustrated in Figure 1.

### 3.2 Basic characteristics for inclusion in the literature

A total of 11 studies were included (18, 20–29), with their basic characteristics summarized in Table 2. The 11 studies enrolled 800 patients, comprising 426 males (53.25%) and 374 females (46.75%). Ten studies were conducted in China, and one in Brazil. All studies provided specific diagnostic criteria, with 10 employing Chinese standards and one using British standards. Within the intervention groups, nine studies utilized body acupuncture, one employed electroacupuncture, and one used scalp electroacupuncture. In the control groups, three studies used Guipi Decoction as the control, four studies used Madopar, one study used Alprazolam, two studies used a combination of two medications, and one study did not specify the control medication. The intervention periods in the 11 studies ranged from 14 days to 8 weeks. Detailed information is presented in Table 2.

**Table 2:** Basic characteristics of included studies.

No	Author	Year	Grouping method	Sample size	Gender (male/female)	Age (±)	Course of the disease	Intervention	Total cycle	Outcome indicators
1	Chen HB. et al.	2021	Merely mentioned randomly	I:31 C:31	I: 18/13 C: 16/15	I: 74.32 ± 5.12 C: 74.54 ± 5.03	I: 4.32 ± 1.21 C:4.27 ± 1.19 y	I: Acupuncture + Guipi Decoction C: Guipi Decoction	I:14d C:14d	Efficacy rates, PSQI, BI Life Skills Assessment
2	Li L. et al.	2021	Random Number Table Method	I:50 C:50	I:27/23 C:26/24	I: 61.56 ± 7.51 C:62.49 ± 7.53	I: 4.08 ± 0.61 C:4.13 ± 0.58 y	I: Electroacupuncture+Levodopa and Benserazide Hydrochloride Tablets C: Levodopa and Benserazide Hydrochloride Tablets	I:8w C:8w	5-HT, DA, BDNF, UPDRS, PDSS, PSQI, SAS, SDS
3	Zhu YC. et al.	2020	Merely mentioned randomly	I:27 C:27	I:16/11 C:17/10	I: 74.2 ± 5.1 C: 73.6 ± 5.3	I:4.5 ± 1 C:4.2 ± 1.1 y	I: Acupuncture + Guipi Decoction C: Guipi Decoction	I:2w C:2w	PSQI, SAS, SDS, BI Life Skills Assessment, Motor dysfunction, Efficacy rates
4	Li L.	2018	Computer software	I:25 C:25	I:13/12 C:13/12	I:69.6 ± 8.9 C:69.7 ± 8.6	/	I: Acupuncture + Guipi Decoction C: Guipi Decoction	I:60d C:60d	PSQI
5	Zhao Y. et al.	2024	Random Number Table Method	I:130 C:130	I:65/65 C:66/64	I:63.44 ± 3.28 C:63.38 ± 3.25	/	I: Acupuncture+Kidney-tonifying and Tremor-relieving Formula combined with Levodopa and Benserazide Hydrochloride Tablets C: Kidney-tonifying and Tremor-relieving Formula combined with Levodopa and Benserazide Hydrochloride Tablets	I:30d C:30d	Efficacy rates, PSQI 5-HT, Dopamine, P-substance, Adverse events
6	Dong QJ. et al.	2018	Random Number Table Method	I:36 C:36	I:19/17 C:21/15	I:60.25 ± 6.376 C: 59.06 ± 7.830	I: 4.03 ± 0.878 C: 3.94 ± 1.013 y	I: Head electroacupuncture C: Levodopa and Benserazide Hydrochloride Tablets	I:30d C:30d	Webster Scale, PDSS, Efficacy rates
7	Li YH. et al.	2019	Random Number Table Method	I:15 C:15	/	/	/	I: Acupuncture C: Levodopa and Benserazide Hydrochloride Tablets	I:4w C:4w	PDSS, PSQI
8	Bai Y. et al.	2021	Order of consultation	I:29 C:29	I:11/18 C:12/17	I:46–78,65.7 C:45–79,64.9	I:6 m-3y, 8.9 m C:6 m-3.2y, 8.8 m	I: Acupuncture+Alprazolam C: Alprazolam	I:2w C:2w	Efficacy rates, PSQI, HAMA
9	Wang Q.	2024	Simple raffle	I:26 C:26	I:14/12 C:15/11	I:66.72 ± 3.21 C:66.40 ± 3.14	I:3.34 ± 0.62 C:3.75 ± 0.69m	I: Levodopa and Benserazide Hydrochloride Tablets+Acupuncture C: Levodopa and Benserazide Hydrochloride Tablets+Agomelatine	I:4w C:4w	PDSS PSQI Total sleep time, Sleep latency, Sleep efficiency, Rapid eye movement sleep duration, Montreal Cognitive Assessment
10	Aroxa FH et al.	2017	Simple raffle	I:11 C:11	I:7/4 C:7/4	I:65 ± 10 C:56 ± 12	/	I: Acupuncture+Pharmacotherapy C: Pharmacotherapy	I:2 m C:2 m	HY scale, MMSE, PDSS
11	Huang N. et al.	2014	Order of consultation	I:20 C:20	I:13/7 C:12/8	I:61 ± 8 C:59 ± 9	I:34 ± 8 C:35 ± 6 m	I: Acupuncture+Levodopa and Benserazide Hydrochloride Tablets C: Levodopa and Benserazide Hydrochloride Tablets	I:4w C:4w	UPDRS, PSQI, Efficacy rates

I, Intervention group; C, Control group; y, year; m, month; d, day.

### 3.3 Quality of studies included

**Figure 2:**
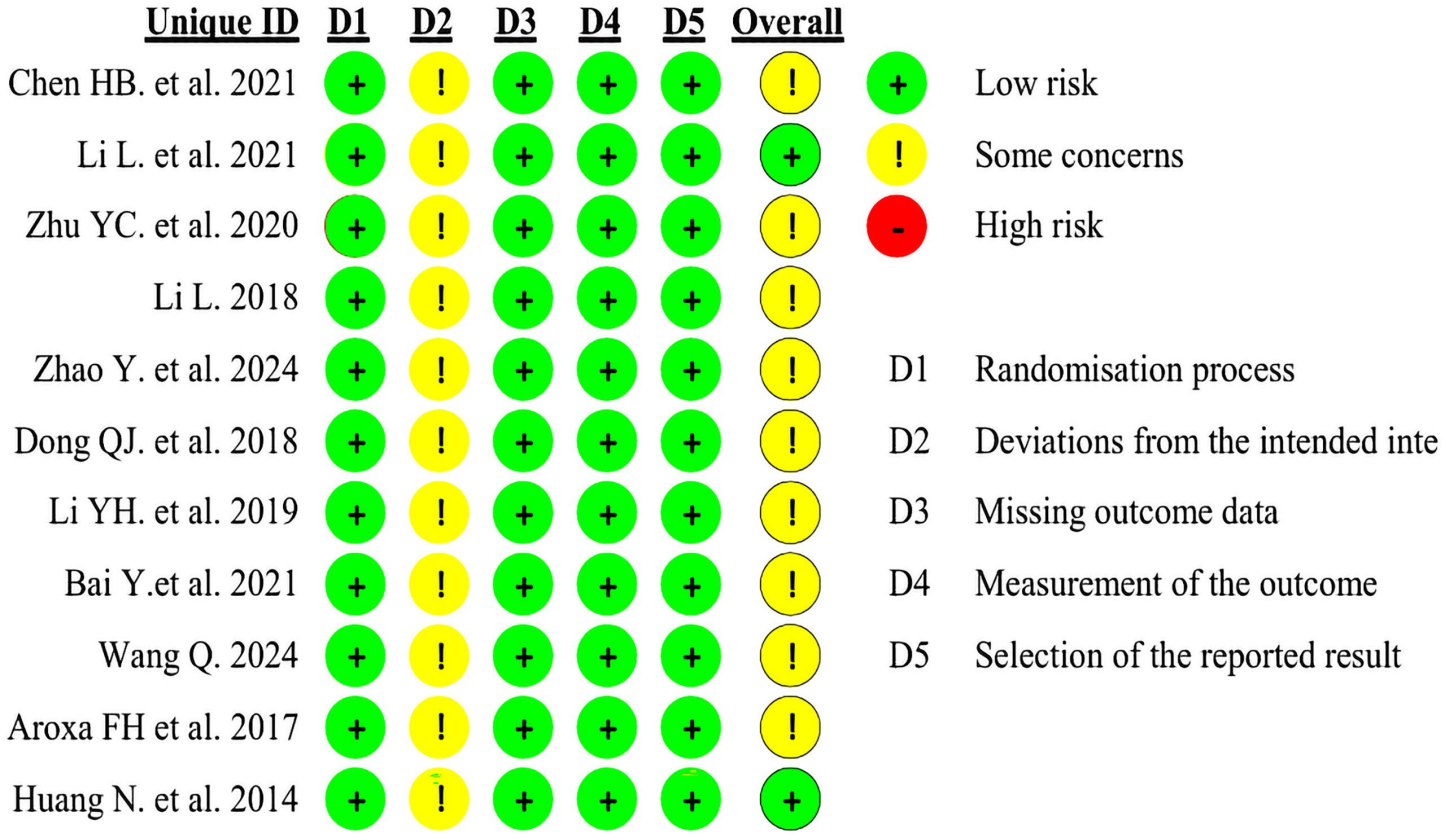
Risk of bias summary.

**Figure 3:**
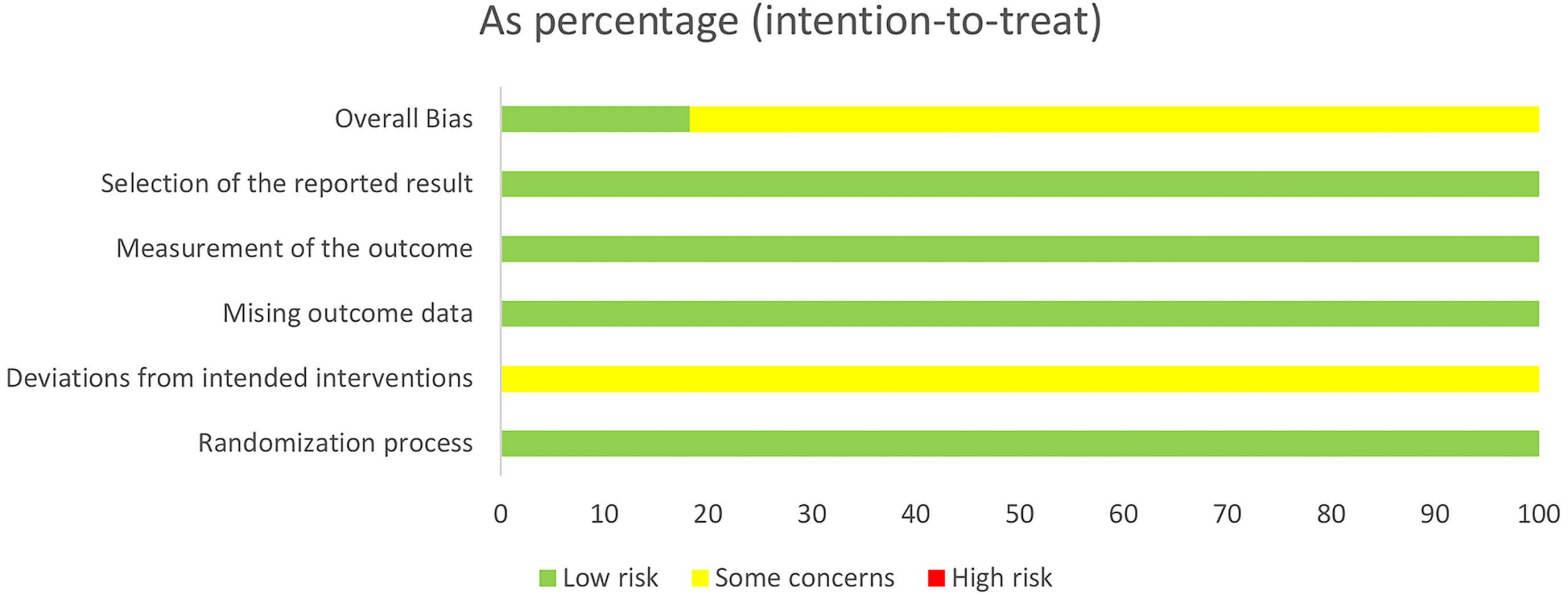
Risk of bias graph.

The included studies were assessed using the Cochrane Risk of Bias tool (ROB 2.0). Four studies employed random number tables for allocation, two studies randomized according to order of presentation, two studies used randomized drawing of lots, two studies merely mentioned random allocation, and one study utilized computer software for allocation. As all trial groups received acupuncture interventions, participant blinding could not be achieved in these studies, thereby compromising their overall quality. Detailed circumstances are presented in Figures 2, 3.

### 3.4 Primary outcome

#### 3.4.1 Pittsburgh sleep quality index

**Figure 4:**
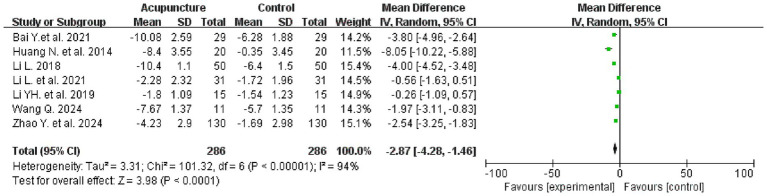
PSQI forest plot.

Seven studies evaluated PSQI scores before and after treatment, involving a total of 572 patients, with 286 patients in each of the treatment and control groups. The meta-analysis revealed that the intervention group demonstrated superiority over the control group in improving PSQI scores [mean difference (MD) = −2.87, 95% confidence interval (CI): −4.28 to −1.46; I^2^ = 94%, *p* < 0.0001] (see Figure 4 for details).

### 3.5 Secondary outcome

#### 3.5.1 Parkinson’s disease sleep scale

**Figure 5:**
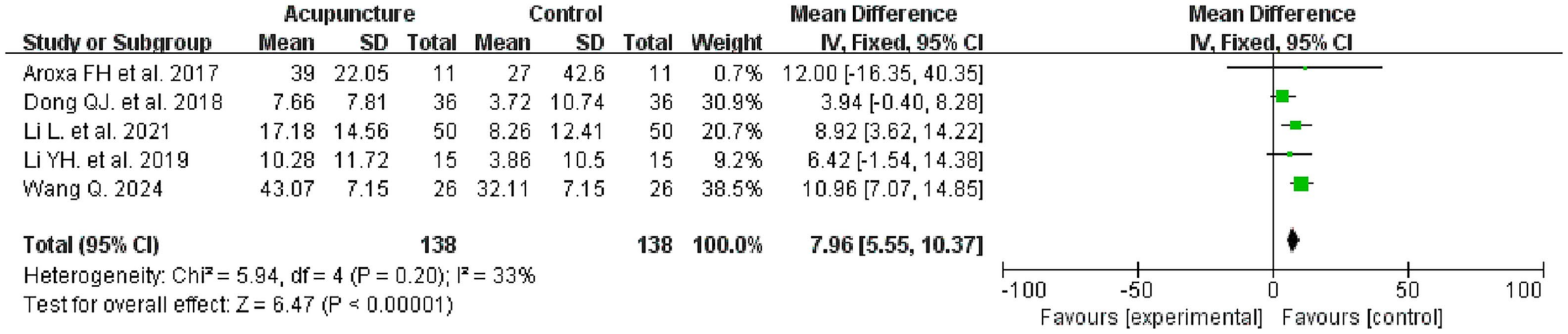
PDSS forest plot.

Five studies evaluated the PDSS before and after treatment, involving 276 patients, with 138 in each of the intervention and control groups. Meta-analysis results indicated that the intervention group demonstrated superior efficacy in improving the PDSS compared to the control group (MD = 7.96, 95% CI: 5.55–10.37, I^2^ = 33%, *p* < 0.00001) (see Figure 5 for details).

#### 3.5.2 Efficacy rates

**Figure 6:**
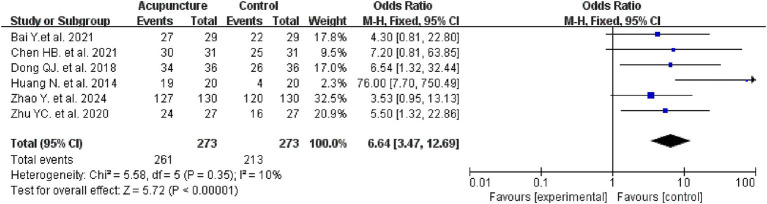
Efficacy rates forest plot.

Six studies evaluated the efficacy rates in two groups, involving 556 patients, with 273 in the intervention group and 273 in the control group. Meta-analysis results indicated that the intervention group demonstrated a higher efficacy rate compared to the control group (OR = 6.64, 95% CI: 3.47–12.69, I^2^ = 10%, *p* < 0.00001) (see Figure 6 for details).

#### 3.5.3 Adverse events

Of the 11 studies, only two mentioned adverse events. Zhao Yun’s study found an adverse reaction rate of 6.15% in the treatment group and 9.23% in the control group, while Dong Qinjian’s study reported adverse reactions occurring in both groups. The remaining nine studies did not mention adverse reactions.

### 3.6 Bias analysis

**Figure 7:**
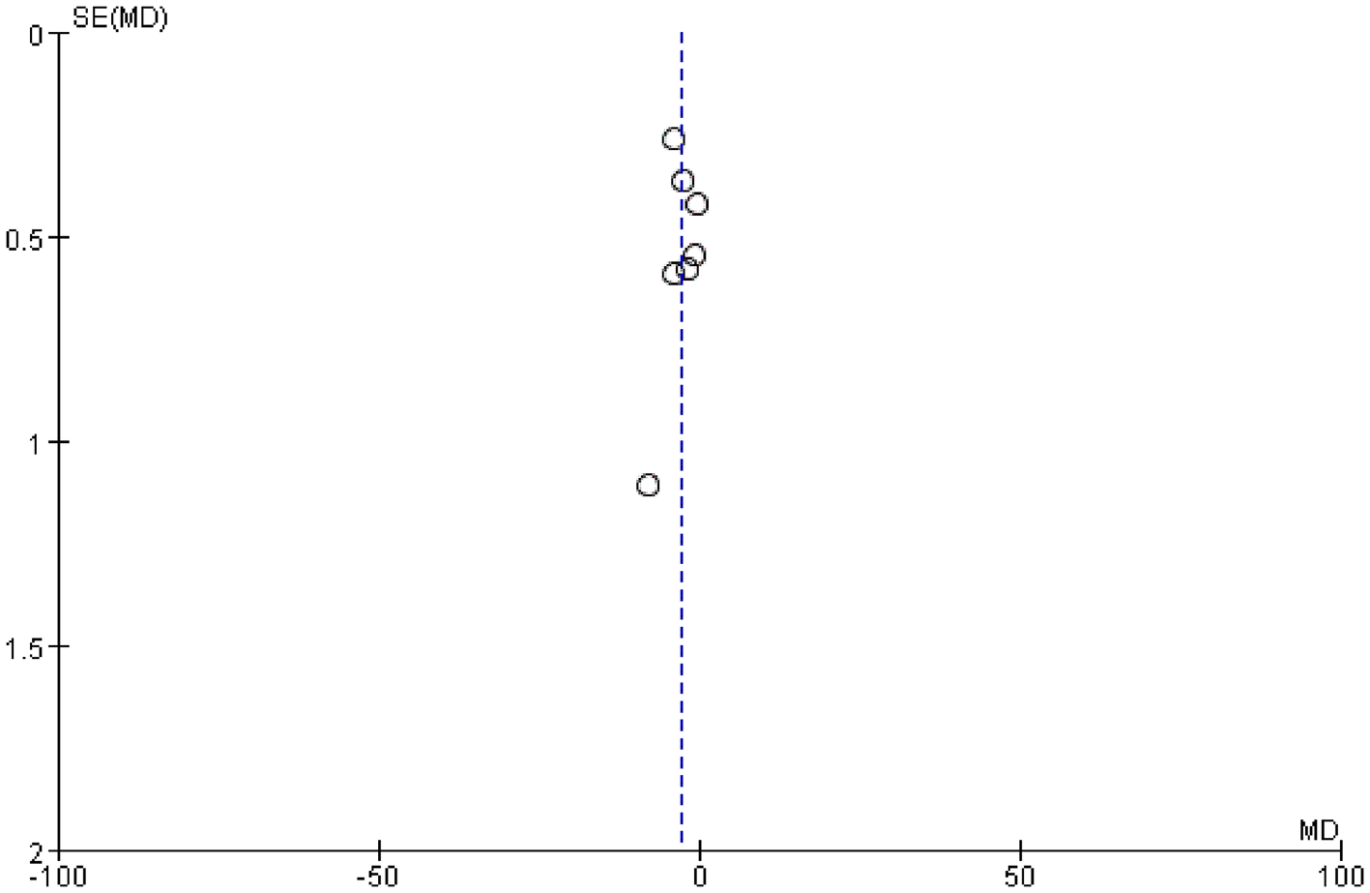
PSQI funnel plot.

We examined whether publication bias existed in this study by plotting a funnel plot; a symmetrical funnel plot indicates no publication bias. Figure 7 presents the funnel plot for the PSQI, revealing no evidence of publication bias in the included studies.

### 3.7 Sensitivity analysis

Observation of the meta-analysis results revealed considerable heterogeneity in the PSQI scores. We conducted a sensitivity analysis by sequentially excluding studies from the analysis using an exclusion method. The exclusion of any single study did not result in a significant change in the heterogeneity. Examination of the relevant studies indicated that five out of six studies had relatively small sample sizes (≤36 participants per group), with only Zhao Yun’s study incorporating a comparatively larger sample size. Consequently, we consider that the heterogeneity may stem from the small sample sizes employed. Further review revealed that the intervention methods included in the studies were body acupuncture, electroacupuncture and scalp acupuncture. The drug interventions received by the control group were also not completely consistent. In addition, there were significant differences in the treatment cycles of each study. All of these may lead to significant heterogeneity.

### 3.8 Evidence level assessment

We employed the GRADE approach to assess the quality of evidence from this study, which yielded moderate-quality evidence. Further details are provided in Supplementary material.

## 4 Discussion

The pathogenesis of PD-related insomnia is multifactorial, primarily involving degeneration of neural structures regulating sleep, alterations in neurotransmitters, or changes in *α*-synuclein (30, 31). As a commonly employed complementary and alternative therapy for psychiatric disorders, acupuncture has been extensively applied in the management of PD-related insomnia (32, 33). Research indicates that acupuncture exerts multi-targeted neuroprotective effects through mechanisms including increased neurotransmitter levels, regulation of α-synuclein, inflammation suppression, and amelioration of cerebral oxidative stress (34–36). Consequently, this study evaluates past clinical evidence via systematic review and meta-analysis to investigate the clinical efficacy and safety of acupuncture for treating PD-related insomnia.

We selected the PSQI as the primary assessment tool, one of the authoritative measures in sleep-related research (37–39), widely employed for evaluating sleep quality in patients with psychiatric and sleep disorders, individuals with various somatic illnesses, and elderly populations (40, 41). Developed through analysis of multiple sleep quality assessment scales, the PSQI evaluates participants’ sleep quality over the preceding month. The PSQI comprises 19 self-report items and 5 observer-report items, with the 19th self-report item and the 5 observer-report items excluded from scoring. The scored items can be grouped into seven components: sleep quality, sleep onset latency, sleep duration, sleep efficiency, and others. The cumulative scores of these components constitute the PSQI total score (0–21 points), where a higher score indicates poorer sleep quality. The PSQI is not only simple to administer but also demonstrates high reliability. Meta-analysis results indicate that acupuncture effectively improves PSQI scores (*p* < 0.0001) compared to control groups, suggesting its efficacy in enhancing sleep. However, it is noteworthy that the included studies only provided pre- and post-treatment PSQI total scores without detailing individual item responses, precluding an assessment of acupuncture’s impact on specific sleep parameters.

We concurrently selected the PD Sleep Scale (PDSS) as one of our assessment tools. This scale represents a multidisciplinary expert-developed criterion for evaluating sleep quality in Parkinson’s disease patients (42). The PDSS comprises 15 common sleep disturbance questions, each scored from 0 to 10. Higher scores indicate better sleep quality. The PDSS not only assesses general sleep patterns but also evaluates the severity of nocturnal symptoms in PD patients (such as limb discomfort, dream disturbances, hallucinations, and tremor upon awakening). Consequently, it provides a more comprehensive assessment of sleep in PD patients and demonstrates high reliability and sensitivity (43). Meta-analysis results indicate that acupuncture effectively improves PDSS scores compared to control groups (*p* < 0.00001), suggesting its advantage in alleviating PD-related insomnia. Similarly, the included studies only provided pre- and post-treatment PDSS total scores, preventing us from understanding acupuncture’s impact on specific components. Meta-analysis of efficacy indicated a higher response rate in the acupuncture group (*p* < 0.00001), suggesting potential and advantages for acupuncture as an adjunctive therapy.

Compared with previous systematic reviews and meta-analyses (44, 45), our study focuses more precisely on the efficacy of acupuncture without interference from other factors. Furthermore, all patients included in the studies incorporated in this research were definitively diagnosed with PD-related insomnia, thereby avoiding the influence of other symptoms on the analysis results. We exclusively included clinical studies published in peer-reviewed journals, ensuring the quality of the included research and enhancing the reliability of the findings.

Overall, acupuncture demonstrated superior efficacy to conventional therapies in improving PSQI, PDSS, and overall effectiveness. Our evidence supports the efficacy of acupuncture as an adjunctive therapy, and the two available studies also appear to indicate its safety. It is noteworthy that, although the findings suggest acupuncture holds certain advantages in treating PD insomnia, no standardized clinical treatment protocol has yet been established. This presents a challenge for further exploration into acupoint selection, treatment frequency, and treatment cycles. Furthermore, the included RCTs did not provide follow-up data, preventing assessment of acupuncture’s long-term efficacy.

## 5 Limitations

In this study, due to the small number of included studies, we were unable to conduct further subgroup analyses to explore the sources of heterogeneity, which made the interpretation of the results cautious. In addition, as only two studies provided data on adverse events, we were unable to accurately assess the safety of acupuncture treatment for this disease. In the future, it is still necessary to strengthen the observation of adverse events during the acupuncture treatment process to obtain reliable data and conclusions.

## 6 Conclusion

The findings of this study indicate that acupuncture effectively improves the PSQI and PDSS scores of PD insomnia patients, demonstrating superior efficacy to conventional therapies. As only two studies provided relevant data on adverse events, we were unable to draw reliable conclusions regarding safety. This conclusion is only applicable to populations with baseline characteristics similar to those of the included studies (such as age, disease duration), and intervention plans.

## Data Availability

The original contributions presented in the study are included in the article/Supplementary material, further inquiries can be directed to the corresponding author.

## References

[1] Tolosa E Garrido A Scholz SW Poewe W. Challenges in the diagnosis of Parkinson’s disease. Lancet Neurol. (2021) 20:385–97. doi: 10.1016/S1474-4422(21)00030-2, PMID: 33894193 PMC8185633

[2] Bloem BR Okun MS Klein C. Parkinson’s disease. Lancet. (2021) 397:2284–303. doi: 10.1016/S0140-6736(21)00218-X, PMID: 33848468

[3] Hou Y Dan X Babbar M Wei Y Hasselbalch SG Croteau DL . Ageing as a risk factor for neurodegenerative disease. Nat Rev Neurol. (2019) 15:565–81. doi: 10.1038/s41582-019-0244-7, PMID: 31501588

[4] Hung AY Schwarzschild MA. Approaches to disease modification for Parkinson’s disease: clinical trials and lessons learned. Neurotherapeutics. (2020) 17:1393–405. doi: 10.1007/s13311-020-00964-w, PMID: 33205384 PMC7851299

[5] Fahn S. The 200-year journey of Parkinson disease: reflecting on the past and looking towards the future. Parkinsonism Relat Disord. (2018) 46:S1–s5. doi: 10.1016/j.parkreldis.2017.07.020, PMID: 28784297

[6] Fearnley JM Lees AJ. Ageing and Parkinson’s disease: substantia nigra regional selectivity. Brain. (1991) 114:2283–301. 1933245 10.1093/brain/114.5.2283

[7] Barber D Wijeratne T Singh L Barnham K Masters CL. The search for disease modification in Parkinson’s disease-a review of the literature. Life. (2025) 15:1169. doi: 10.3390/life15081169, PMID: 40868817 PMC12387364

[8] Falup-Pecurariu C Diaconu Ş. Sleep dysfunction in Parkinson’s disease. Int Rev Neurobiol. (2017) 133:719–42. doi: 10.1016/bs.irn.2017.05.033, PMID: 28802939

[9] Thangaleela S Sivamaruthi BS Kesika P Mariappan S Rashmi S Choeisoongnern T . Neurological insights into sleep disorders in Parkinson’s disease. Brain Sci. (2023) 13:1202. doi: 10.3390/brainsci13081202, PMID: 37626558 PMC10452387

[10] Taximaimaiti R Luo X Wang XP. Pharmacological and non-pharmacological treatments of sleep disorders in Parkinson’s disease. Curr Neuropharmacol. (2021) 19:2233–49. doi: 10.2174/1570159X19666210517115706, PMID: 33998990 PMC9185775

[11] Falup-Pecurariu C Muntean ML Ungureanu L Murasan I Popławska-Domaszewicz K Chaudhuri KR . Pharmacological and non-pharmacological management of sleep disturbances in Parkinson’s disease: if when and how. Expert Opin Pharmacother. (2024) 25:2135–49. doi: 10.1080/14656566.2024.2422004, PMID: 39535843

[12] Stefani A Högl B. Sleep in Parkinson’s disease. Neuropsychopharmacology. (2020) 45:121–8. doi: 10.1038/s41386-019-0448-y, PMID: 31234200 PMC6879568

[13] Suzuki K Fujita H Kobayashi S. Managing sleep issues in Parkinson’s disease: an up-to-date review. Expert Rev Neurother. (2025) 25:211–26. doi: 10.1080/14737175.2025.2450789, PMID: 39789992

[14] Cheng FK. The use of acupuncture in patients with Parkinson’s disease. Geriatric Nurs. (2017) 38:302–14. doi: 10.1016/j.gerinurse.2016.11.010, PMID: 28041638

[15] Wu WZ Zheng SY Liu CY Qin S Wang XQ Hu JL . Effect of Tongdu Tiaoshen acupuncture on serum GABA and CORT levels in patients with chronic insomnia. Zhongguo zhen jiu. (2021) 41:721–4. doi: 10.13703/j.0255-2930.20200704-k0001, PMID: 34259401

[16] Li YW Li W Wang ST Gong YN Dou BM Lyu ZX . The autonomic nervous system: a potential link to the efficacy of acupuncture. Front Neurosci. (2022) 16:1038945. doi: 10.3389/fnins.2022.1038945, PMID: 36570846 PMC9772996

[17] Cao F Xu Y Zhang M Li X Chen Y Zhi M . Baihui (DU20), Shenmen (HT7) and Sanyinjiao (SP6) target the cAMP/CREB/BDNF and PI3K/Akt pathways to reduce central nervous system apoptosis in rats with insomnia. Heliyon. (2022) 8:e12574. doi: 10.1016/j.heliyon.2022.e12574, PMID: 36636219 PMC9830165

[18] Aroxa FH Gondim IT Santos EL Coriolano MD Asano AG Asano NM. Acupuncture as adjuvant therapy for sleep disorders in Parkinson’s disease. J Acupunct Meridian Stud. (2017) 10:33–8. doi: 10.1016/j.jams.2016.12.007, PMID: 28254099

[19] Huang CH Lin SK Lin MC Hung SY. Reducing Parkinson’s disease incidence in patients with insomnia through acupuncture: a cohort study. Int Med Res. (2024) 13:101083. doi: 10.1016/j.imr.2024.101083, PMID: 39635074 PMC11616590

[20] Bai Y Wang M. Clinical observation of 56 cases of acupuncture treatment for Parkinson’s disease with insomnia. Chin J Tradit Med Sci Technol. (2021) 28:506–7.

[21] Chen H. Clinical observation on the combined use of modified Guipi decoction and acupuncture for insomnia in Parkinson’s disease with deficiency of both heart and spleen. World Latest Med Inform. (2021) 21:469–70.

[22] Dong Q Li X Tang M Wan J Zhong L. A randomized controlled trial of transcranial direct current stimulation for sleep disorders in Parkinson’s disease. Practi J Clin Med. (2018) 15:214–6.

[23] Huang N Huang L An J. Clinical observation of fang’s scalp acupuncture in treating insomnia associated with Parkinson’s disease. Shaanxi J Tradit Chin Med. (2014) 35:348–9.

[24] Li L. Clinical observation on Guipi decoction combined with acupuncture and Moxibustion in the treatment of insomnia patients with Parkinson. Guangming Journal of Chinese Medicine. (2018) 33:2043–4.

[25] Li L Tian Z Zhang X. Clinical study on electroacupuncture combined with Madopar for sleep disorders in Parkinson disease. New Chinese Med. (2021) 53:113–6.

[26] Li Y Wang Y Zhan X Zhao Y Wu H Wang H. 15 cases of sleep disorder of Parkinson’s disease treated with acupuncture combined with psychotherapy. J Jiangxi Univ Chin Med. (2019) 31:55–7.

[27] Wang Q. Clinical efficacy study of acupuncture treatment for sleep disorders in Parkinson’s disease. Chin Sci Technol J Database. (2024) 12:103–106.

[28] Zhao Y Li M Jie Z. Effects of Tongdu Jieyu acupuncture combined with Bushen Zhichan prescription and levodopa and Benserazide hydrochloride tablets in treatment of patients with Parkinson’s disease with sleep disorders. Med J Chin Peoples Health. (2024) 36:82–5.

[29] Zhu Y Ling W. Clinical observation on the treatment of insomnia in Parkinson’s disease with heart-spleen deficiency pattern using modified Guipi decoction combined with acupuncture. Guangming J Chin Med. (2020) 35:3253–5.

[30] Wang XT Yu H Liu FT Zhang C Ma YH Wang J . Associations of sleep disorders with cerebrospinal fluid α-synuclein in prodromal and early Parkinson’s disease. J Neurol. (2022) 269:2469–78. doi: 10.1007/s00415-021-10812-2, PMID: 34605986

[31] Schapira AHV Chaudhuri KR Jenner P. Non-motor features of Parkinson disease. Nat Rev Neurosci. (2017) 18:435–50. doi: 10.1038/nrn.2017.62, PMID: 28592904

[32] Li M Yang X Jiang L Yang D. Acupuncture for rapid eye movement sleep behavior disorder in Parkinson’s disease: a case report. Acupunct Med. (2022) 40:203–4. doi: 10.1177/09645284211041125, PMID: 34886712

[33] Zang Z Yang F Qu L Ge M Tong L Xue L . Acupuncture modulates the microbiota-gut-brain axis: a new strategy for Parkinson’s disease treatment. Front Aging Neurosci. (2025) 17:1640389. doi: 10.3389/fnagi.2025.1640389, PMID: 40851666 PMC12367693

[34] Yeo S Lim S. Acupuncture inhibits the increase in alpha-Synuclein by modulating SGK1 in an MPTP induced parkinsonism mouse model. Am J Chin Med. (2019) 47:527–39. doi: 10.1142/S0192415X19500277, PMID: 30966771

[35] Zuo T Xie M Yan M Zhang Z Tian T Zhu Y . In situ analysis of acupuncture protecting dopaminergic neurons from lipid peroxidative damage in mice of Parkinson’s disease. Cell Prolif. (2022) 55:e13213. doi: 10.1111/cpr.13213, PMID: 35274781 PMC9055900

[36] Jang JH Yeom MJ Ahn S Oh JY Ji S Kim TH . Acupuncture inhibits neuroinflammation and gut microbial dysbiosis in a mouse model of Parkinson’s disease. Brain Behav Immun. (2020) 89:641–55. doi: 10.1016/j.bbi.2020.08.015, PMID: 32827699

[37] Buysse DJ Ancoli-Israel S Edinger JD Lichstein KL Morin CM. Recommendations for a standard research assessment of insomnia. Sleep. (2006) 29:1155–73. doi: 10.1093/sleep/29.9.1155, PMID: 17040003

[38] Hartmann JA Carney CE Lachowski A Edinger JD. Exploring the construct of subjective sleep quality in patients with insomnia. J Clin Psychiatry. (2015) 76:e768–73. doi: 10.4088/JCP.14m09066, PMID: 26132684

[39] Buysse DJ Reynolds CF 3rd Monk TH Berman SR Kupfer DJ. The Pittsburgh sleep quality index: a new instrument for psychiatric practice and research. Psychiatry Res (1989);28:193–213. doi: 10.1016/0165-1781(89)90047-4 2748771

[40] Carpenter JS Andrykowski MA. Psychometric evaluation of the Pittsburgh sleep quality index. J Psychosom Res. (1998) 45:5–13. doi: 10.1016/S0022-3999(97)00298-5, PMID: 9720850

[41] Sateia MJ Doghramji K Hauri PJ Morin CM. Evaluation of chronic insomnia. An American Academy of sleep medicine review. Sleep. (2000) 23:243–308. doi: 10.1093/sleep/23.2.1l, PMID: 10737342

[42] Chaudhuri KR Pal S DiMarco A Whately-Smith C Bridgman K Mathew R . The Parkinson’s disease sleep scale: a new instrument for assessing sleep and nocturnal disability in Parkinson’s disease. J Neurol Neurosurg Psychiatry. (2002) 73:629–35. doi: 10.1136/jnnp.73.6.629, PMID: 12438461 PMC1757333

[43] Högl B Arnulf I Comella C Ferreira J Iranzo A Tilley B . Scales to assess sleep impairment in Parkinson’s disease: critique and recommendations. Mov Disord. (2010) 25:2704–16. doi: 10.1002/mds.23190, PMID: 20931631

[44] Yan F Chen C Feng Q Huang Z Chen Y Chen H. Acupuncture and sleep disorders in Parkinson’s disease: a systematic evaluation with meta-analysis. Medicine. (2024) 103:e36286. doi: 10.1097/MD.0000000000036286, PMID: 38181255 PMC10766232

[45] Mi W Meng M Xu F Sun L. Efficacy of acupuncture as adjunct therapy for sleep disorders in Parkinson’s disease: a systematic review and meta-analysis. Complement Ther Med. (2024) 82:103044. doi: 10.1016/j.ctim.2024.103044, PMID: 38679147

