# Supplementary material for: The efficacy and safety of acupuncture for Parkinson’s disease insomnia: a systematic review and meta-analysis
Source: Front Neurol. 2025 Nov 3;16:1697481. doi: 10.3389/fneur.2025.1697481 (PMC12620196; doi:10.3389/fneur.2025.1697481)
Supplement: Supplementary file 1 [file Table_1.docx]

**Author(s):**

**Question:** Acupuncture compared to control for Parkinson's-related insomnia

**Setting:**

**Bibliography:** . Acupuncture for Parkinson's-related insomnia. Cochrane Database of Systematic Reviews [Year], Issue [Issue].

| **Certainty assessment** | | | | | | | **№ of patients** | | **Effect** | | **Certainty** | **Importance** |
| --- | --- | --- | --- | --- | --- | --- | --- | --- | --- | --- | --- | --- |
| **№ of studies** | **Study design** | **Risk of bias** | **Inconsistency** | **Indirectness** | **Imprecision** | **Other considerations** | **针刺治疗帕金森失眠** | **placebo** | **Relative (95% CI)** | **Absolute (95% CI)** |  |  |
| **PSQI** | | | | | | | | | | | | |
| 7 | randomised trials | serious^a^ | not serious | not serious | not serious | none | 286 | 286 | - | MD **2.87 lower** (4.28 lower to 1.46 lower) | ⨁⨁⨁◯ Moderate^a^ |  |
| **PDSS** | | | | | | | | | | | | |
| 5 | randomised trials | serious^a^ | not serious | not serious | not serious | none | 138 | 138 | - | MD **7.96 higher** (5.55 higher to 10.37 higher) | ⨁⨁⨁◯ Moderate^a^ |  |
| **有效率** | | | | | | | | | | | | |
| 6 | randomised trials | serious^a^ | not serious | not serious | not serious | none | 261/273 (95.6%) | 213/273 (78.0%) | **OR 6.64** (3.47 to 12.69) | **179 more per 1,000** (from 145 more to 198 more) | ⨁⨁⨁◯ Moderate^a^ |  |

**CI:** confidence interval; **MD:** mean difference; **OR:** odds ratio

#### Explanations

a. Since acupuncture was used as the intervention in the study and patients signed informed consent forms, it was not possible to achieve complete blinding.
